# Cancer Associated Fibroblasts Promote Tumor Growth and Metastasis by Modulating the Tumor Immune Microenvironment in a 4T1 Murine Breast Cancer Model

**DOI:** 10.1371/journal.pone.0007965

**Published:** 2009-11-23

**Authors:** Debbie Liao, Yunping Luo, Dorothy Markowitz, Rong Xiang, Ralph A. Reisfeld

**Affiliations:** 1 Department of Immunology and Microbial Science, The Scripps Research Institute, La Jolla, California, United States of America; 2 Department of Immunology, Nankai University School of Medicine, Tianjing, China; Bauer Research Foundation, United States of America

## Abstract

**Background:**

Local inflammation associated with solid tumors commonly results from factors released by tumor cells and the tumor stroma, and promotes tumor progression. Cancer associated fibroblasts comprise a majority of the cells found in tumor stroma and are appealing targets for cancer therapy. Here, our aim was to determine the efficacy of targeting cancer associated fibroblasts for the treatment of metastatic breast cancer.

**Methodology/Principal Findings:**

We demonstrate that cancer associated fibroblasts are key modulators of immune polarization in the tumor microenvironment of a 4T1 murine model of metastatic breast cancer. Elimination of cancer associated fibroblasts *in vivo* by a DNA vaccine targeted to fibroblast activation protein results in a shift of the immune microenvironment from a Th2 to Th1 polarization. This shift is characterized by increased protein expression of IL-2 and IL-7, suppressed recruitment of tumor-associated macrophages, myeloid derived suppressor cells, T regulatory cells, and decreased tumor angiogenesis and lymphangiogenesis. Additionally, the vaccine improved anti-metastatic effects of doxorubicin chemotherapy and enhanced suppression of IL-6 and IL-4 protein expression while increasing recruitment of dendritic cells and CD8^+^ T cells. Treatment with the combination therapy also reduced tumor-associated Vegf, Pdgfc, and GM-CSF mRNA and protein expression.

**Conclusions/Significance:**

Our findings demonstrate that cancer associated fibroblasts promote tumor growth and metastasis through their role as key modulators of immune polarization in the tumor microenvironment and are valid targets for therapy of metastatic breast cancer.

## Introduction

The solid tumors of multiple cancers, including that of the breast, are often associated with local inflammation [1]. In fact, cancer-related inflammation has recently been proposed as the seventh hallmark of cancer [2]. During normal physiological processes, such as wound healing, inflammatory cells recruited to the site of injury support tissue repair through secretion of growth factors and cytokines that promote tissue remodeling and angiogenesis [3]. Importantly, inflammation subsides once the tissue has been repaired [3]. In contrast, the normal controls regulating inflammation are circumvented during neoplastic progression which has resulted in the characterization of tumors as wounds that never heal [4].

Solid tumors are multi-cellular tissues comprised of tumor cells and stromal cells, including fibroblasts, endothelial cells and inflammatory cells [5]. Recent studies focusing on cancer-associated fibroblasts (CAFs) have begun to uncover their prominent role in promoting tumor growth and progression [6]. In contrast to resting fibroblasts, CAFs possess an activated phenotype and can be identified by their expression of vimentin, desmin, α-smooth-muscle actin and fibroblast activation protein (FAP) [7]. CAFs are further characterized by their production of growth factors and extracellular matrix proteins that promote proliferation and survival of tumor cells [6]. Additionally, up to 80% of stromal fibroblasts in breast cancer are thought to possess this activated phenotype [6]. However, compared to transformed tumor cells, CAFs are considerably more genetically homogeneous and thus represent an attractive target for cancer therapy [8].

We have previously reported that a DNA vaccine targeted to Fibroblast Activation Protein (pFAP) can specifically and effectively eliminate CAFs *in vivo* and suppress primary tumor growth of non-metastatic murine colon and breast cancers [9]. FAP, also known as Seprase, is a type II transmembrane glycoprotein belonging to the serine protease family and was first isolated from human malignant melanoma [10]. FAP expression has since been observed in more than 90% of all human epithelial tumors, including breast carcinomas, and their metastases [11]. Additionally, the expression pattern of FAP was shown to be highly cancer-specific and localized to stromal fibroblasts in solid tumors [12]. Similarly, we have also observed stroma-specific expression of FAP in murine carcinomas [9]. Importantly, we have also previously shown that vaccination of mice with pFap did not impair wound healing or cause toxicity in normal tissues [9].

In the current study our aim was to determine the efficacy of our vaccine for suppression of spontaneous breast cancer metastasis and to further investigate the broader cellular effects of our vaccine on the tumor microenvironment (TME). Our finding here demonstrate that CAFs are key modulators of the immune TME and that their elimination *in vivo* has profound effects on immune polarization in the TME. Importantly, this modulation of immune polarization is associated with decreased tumor angiogenesis, lymphangiogensis, and suppression of spontaneous breast cancer metastasis.

## Results

### Elimination of CAFs Suppresses Spontaneous Metastasis and Enhances the Anti-Metastatic Effects of Chemotherapy

**Figure 1:**
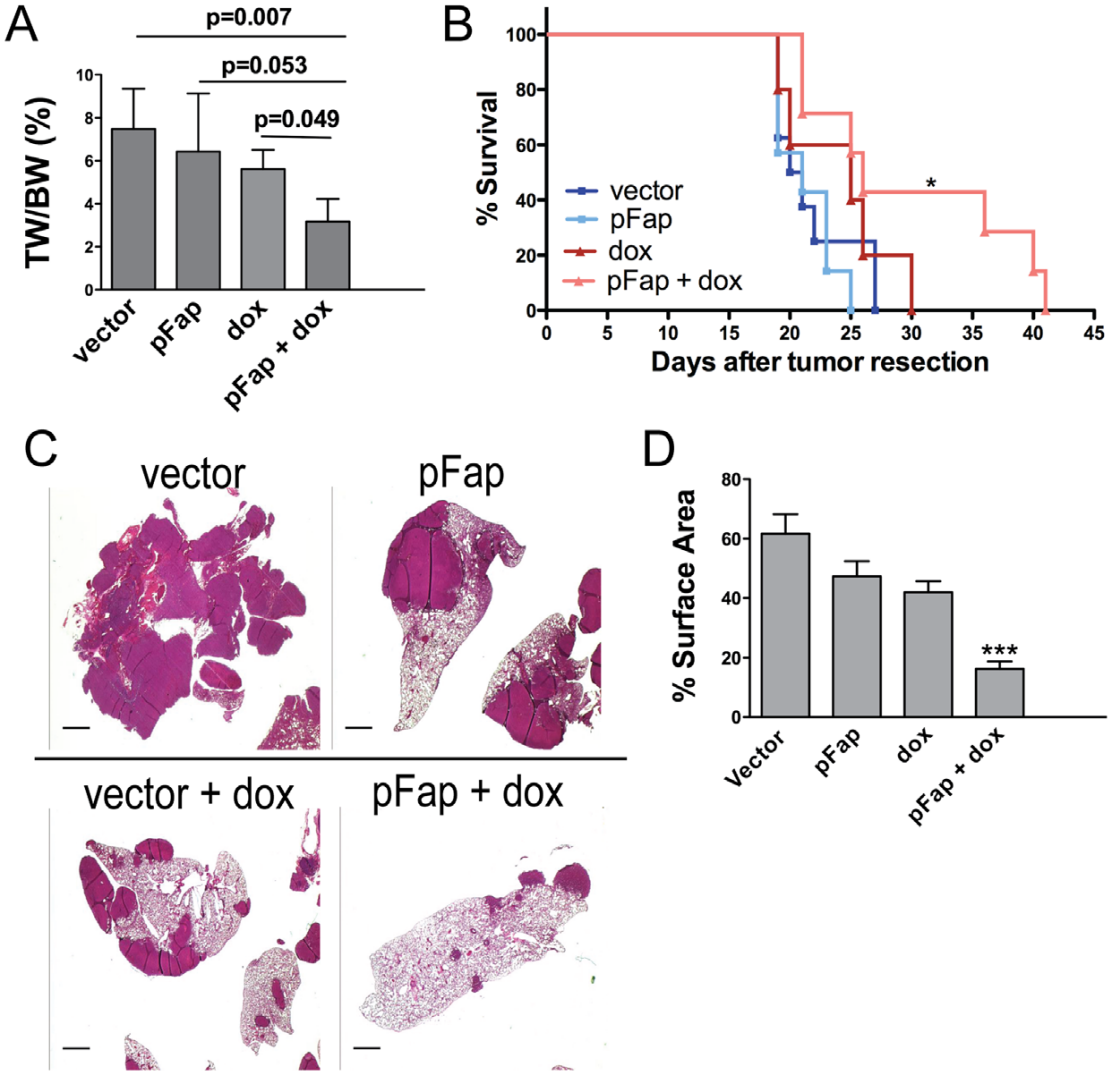
Vaccination with pFap enhances the anti-metastaic effects of doxorubicin chemotherapy. (A) Mice were treated in a prophylactic setting and sacrificed 25 days after tumor cell challenge. Primary tumors were resected and their weight (TW) was compared to body weight (BW) to calculate tumor burden. (B) Mice were treated in a therapeutic setting where primary tumors were allowed to establish prior to resection and treatment with the combination therapy. Survival of mice after tumor resection was measured using Kaplan-Meier survival curves. *, p<0.05. (C) Lungs were isolated from moribund mice treated in a therapeutic setting. To visualize metastatic foci, lungs were sectioned and stained with hematoxylin and eosin. Scale bar = 1 mm. (D) Surface areas (SA) of metastatic foci and lung were measured using ImageJ software (n = 5 mice/group). Results are depicted as percent SA^metastasis^/SA^lung^. ***, p<0.0005.

To evaluate the efficacy of pFap vaccination for suppressing spontaneous breast cancer metastasis, immune competent Balb/c mice were challenged orthotopically with 4T1 tumor cells and vaccinated in either a prophylactic (before tumor cell challenge) or therapeutic (after tumor cell challenge) setting. Additionally, we also investigated the effects of pFap vaccination when combined with doxorubicin, a chemotherapeutic drug commonly used in breast cancer. Prophylactic vaccination with pFap significantly enhanced the anti-tumor effects of doxorubicin chemotherapy and suppressed the growth of 4T1 primary tumors *in vivo*, confirming our previous findings in non-metastatic tumor models (Figure 1A). Next, we examined the effect on spontaneous metastasis by treating mice in a clinically relevant therapeutic model where therapy was administered after resection of an established primary tumor. In this therapeutic setting, the addition of pFap vaccination to doxorubicin chemotherapy significantly increased the life span of mice with spontaneous metastatic disease (Figure 1B). Additionally, hematoxylin and eosin staining of lungs from treated mice showed that the pFap vaccine markedly enhanced the anti-metastatic effects of doxorubicin chemotherapy as evidenced by the reduction in metastatic foci (Figure 1C). In contrast, either pFap vaccination or chemotherapy alone only modestly suppressed spontaneous metastasis though neither was as effective as the combination therapy. A comparison of the respective metastatic surface areas revealed that our combination therapy significantly reduced metastatic load compared to all control groups (Figure 1D). These data demonstrate that eliminating CAFs with our pFap vaccine suppresses spontaneous metastasis and markedly enhances the anti-metastatic effects of doxorubicin chemotherapy in a 4T1 murine breast cancer model.

### Th2 to Th1 Cytokine Transition, CTL Activation and Tumor Cell Killing in Primary Tumors Following Combination Therapy

**Figure 2:**
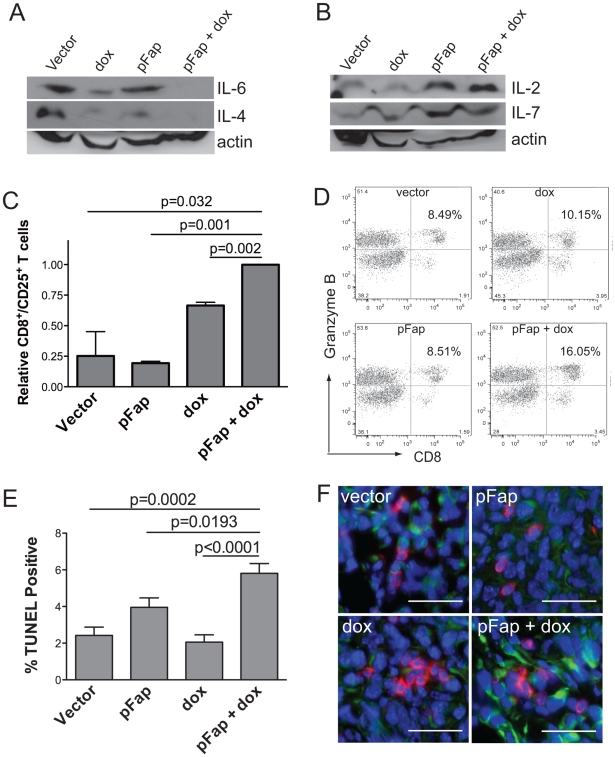
Vaccination with pFap results in Th2 to Th1 cytokine transition in the tumor microenvironment. Mice were treated in a prophylactic setting and primary tumors isolated 25 days later. (A–B) Tumor homogenates were analyzed by Western blotting to determine IL-6 and IL4 (A) and IL-2 and IL-7 (B) protein levels. (C) Live primary tumor cell suspensions were analyzed by flow cytometry using anti-CD8 and anti-CD25 antibodies to detect activated T-cells. Results are shown as percent of CD8^+^/CD25^+^ T cells relative to mice treated with the combination therapy. (D) Splenocytes were isolated from tumor bearing mice treated with the combination therapy or controls. Splenocytes were stimulated twice with IL-2 and then incubated with irradiated 4T1 tumor cells for 24 hours prior to detection of CD8^+^/Granzyme B^+^ cells by flow cytometry. (E) Primary tumors were isolated from mice treated in a prophylactic setting 10-days after the final treatment with doxorubicin and apoptotic tumor cells were visualized by quantified using the TUNEL assay. (F) Additionally, we also used immunohistochemistry to visualize CD8^+^ T cells (red) and apoptotic tumor cells expressing caspase 3 (green) in primary tumors. Scale bar = 100 µm.

Cell-mediated immunity is associated with Th1 polarization and generally favors tumor rejection as a result of anti-tumor CTL responses [13]. In contrast, Th2 cytokine polarization, which is most commonly associated with solid tumors, generally prevents tumor rejection and promotes tumor growth [13]. Since Th1 versus Th2 polarization is determined in part by cytokines in the TME, we investigated the expression of known Th1 and Th2 cytokines by immunoblotting of whole tumor extracts. Western blotting showed that pFap vaccination markedly increased IL-2 and IL-7 Th1 cytokine expression in primary tumors (Figure 2B). In contrast, the doxorubicin-induced reduction of IL-6 and IL-4 Th2 cytokine expression in the TME was enhanced by pFap vaccination (Figure 2A).

Analysis of live tumor homogenates by flow cytometry revealed that the shift from Th2 to Th1 cytokine expression correlated with a significant increase in the relative percentage of activated CD8^+^/CD25^+^ T cells in the TME (Figure 2C). Additionally, CD8^+^ T cells isolated from spleens of mice treated with our combination therapy showed enhanced Granzyme B production following stimulation with irradiated 4T1 cells *ex vivo*, thus demonstrating an improved anti-tumor CTL memory effect (Figure 2D). TUNEL immunohistochemistry, performed on primary tumor sections also revealed that our combination therapy resulted in significant increases in the percentage of apoptotic tumor cells 16-days after cessation of treatment (Figure 2E). Further immunohistochemical analysis of primary tumors also revealed a marked increase in association between CD8^+^ T cells and caspase 3^+^ tumor cells following treatment with the combination therapy (Figure 2F). Collectively, these results demonstrate that the pFap vaccine, when combined with doxorubicin chemotherapy, results in a shift in cytokine polarization in the TME from Th2 to Th1 and consequently enhances CTL-associated tumor cell killing in primary breast tumors.

### Elimination of CAFs Reduces Recruitment of Immune Suppressor Cells and Enhances Immune Effector Cell Recruitment to Primary Tumors

**Figure 3:**
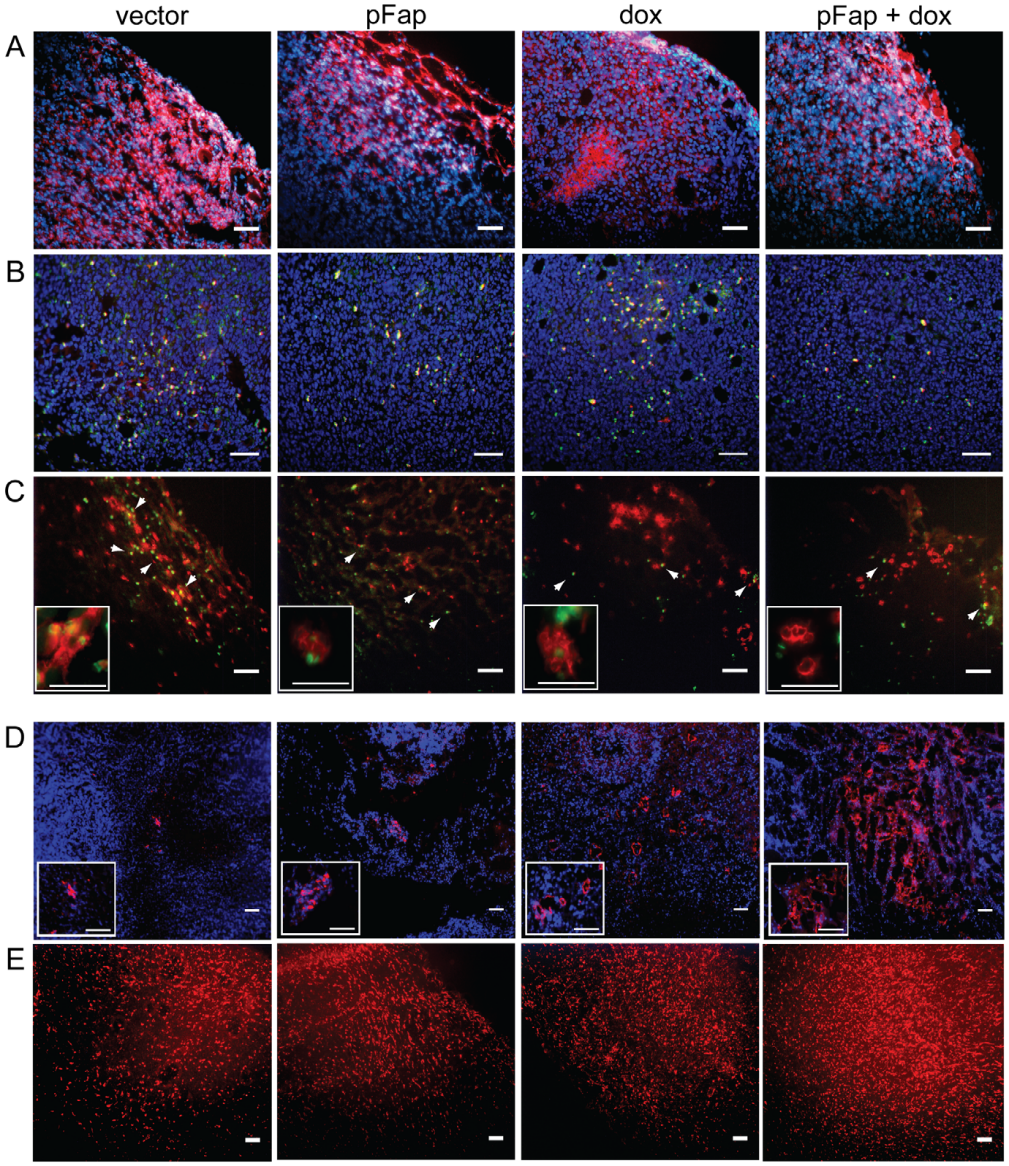
Vaccination with pFap suppresses TAM, MDSC, Treg, and enhances DC and CTL recruitment. Primary tumors were isolated from mice 25 days after orthotopic challenge and immune cells were identified in primary tumors using antibodies for the following: (A) tumor-associated macrophages (TAMs): F4/80 (red), (B) myeloid derived suppressor cells (MDSCs): CD11b (red, nuclear) and Gr-1 (green, nuclear), (C) T regulatory cells (Tregs, white arrowheads): CD4 (red, cell surface) and FOXP3 (green, nuclear), (D) dendritic cells (DC): 33D1 (red), and (E) cytotoxic T lymphocytes (CTL): CD8 (red) Scale bar for all panels = 100 µm.

Tumor infiltrating immune cells such as tumor associated macrophages (TAMs) and myeloid derived suppressor cells (MDSCs) are key producers of Th2 cytokines and other factors that suppress host anti-tumor immune responses while promoting tumor growth [2]. To determine if the shift from Th2 to Th1 cytokine polarization induced by our combination therapy correlated with changes in the immune cell milieu of the TME, we used immunohistochemistry to identify tumor associated macrophages (TAMs), myeloid derived suppressor cells (MDSCs), T regulatory cells (Tregs), dendritic cells (DCs) and CD8^+^ T cells in primary tumors. Importantly, vaccination with pFap markedly reduced the staining in the TME for F4/80^+^ TAMs (Figure 3A), CD11b^+^/Gr-1^+^ MDSCs (Figure 3B), and CD4^+^/FOXP3^+^ Tregs (Figure 3C). In contrast, pFap vaccination markedly enhanced doxorubicin-associated increases in staining for DCs (Figure 3D) and CD8^+^ T cells (Figure 3E) in the TME. These results demonstrate that CAFs are key modulators of the immune cell milieu in the TME and vaccination with pFap can effectively suppress recruitment of pro-tumor immune cells while enhancing chemotherapy-induced recruitment of anti-tumor immune cells.

### Combination Therapy Inhibits Tumor Angiogenesis and Lymphangiogenesis and Reduces Vegf, Pdgfc, and GM-CSF Expression by the TME

**Figure 4:**
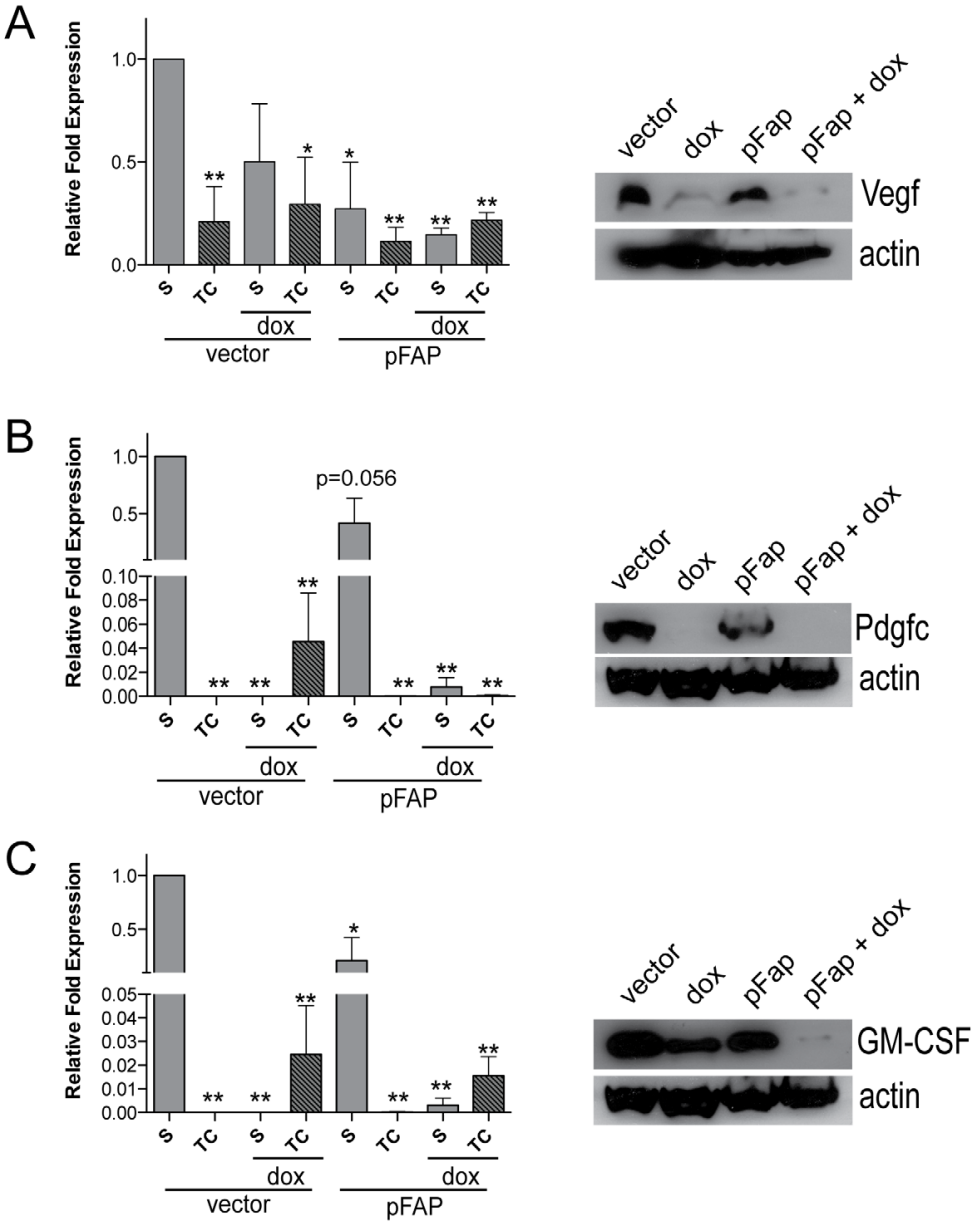
Combination therapy reduces tumor associated Vegf, Pdgfc, and GM-CSF mRNA and protein expression. Tumors were isolated 25 days after orthothopic challenge and total RNA was isolated from the stroma (S) and tumor cells (TC) by laser capture dissection microscopy, and used to generate cDNA for qRT-PCR analysis. Gene expression is normalized to actin and shown relative to stroma of vector control for Vegfa, Pdgfc, and GM-CSF (A–C, left panel, respectively). *, p<0.05, **, p<0.005. Whole cell extracts were derived from primary tumors and subjected to immunoblotting to detect Vegf, Pdgfc and GM-CSF protein and detection of actin was used as a loading control (A–C, right panel, respectively).

Cells prominent in the tumor stroma, including CAFs and TAMs, produce growth factors and cytokines that support tumor growth and facilitate metastasis by promoting angiogenesis [14], [15], [16]. Therefore, we next determined the effects of pFap vaccine-induced immune modulation on the expression of pro-angiogenesis factors in the TME. First, qRT-PCR analysis of stroma and tumor cell mRNA from primary tumors showed that the relative expression of Vegfa (Figure 4A), Pdgfc (Figure 4B) and GM-CSF (Figure 4C) was highest in the tumor stroma. However, the expression of these growth factors was reduced by pFap vaccination. In concordance with our mRNA data, Western blot analysis of tumor extracts demonstrated that the protein level of these factors was also markedly reduced by our combination therapy (Figure 4A–C). Additionally, pFap vaccination distinctly enhanced the chemotherapy-induced reduction of both VEGF as well as GM-CSF mRNA and protein expression.

**Figure 5:**
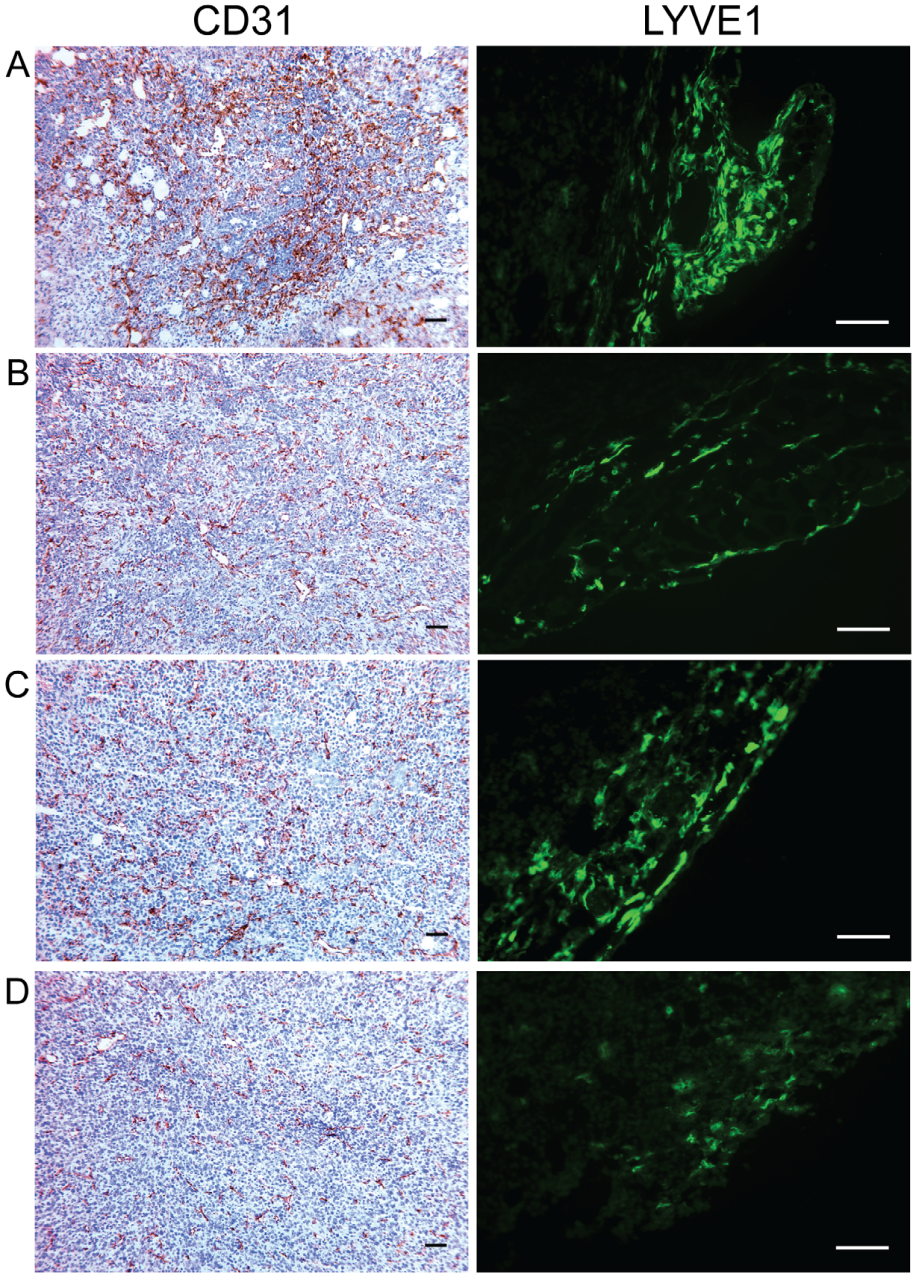
Vaccination with pFap suppresses tumor angiogenesis and lymphangiogenesis. Mice were treated with vector control (A), pFap (B), doxorubicin (C), or pFap plus doxorubicin (D). Primary tumors were isolated from mice 25 days after orthotopic challenge. Tumor sections were treated with anti-CD31 and anti-LYVE1 antibodies to detect blood (brown) and lymph (green) vessels, respectively. Scale bar = 100 µm.

Finally, we determined that differences in Vegf, Pdgf, and GM-CSF gene expression induced by our combination therapy resulted in changes to tumor vascularization. Blood and lymph vessels were visualized by immunohistochemistry with CD31 and LYVE1 antibodies, respectively, and staining for both was greatly reduced in primary tumors by pFap vaccination (Figure 5). These results demonstrate that immune modulation of the TME as a result of pFap vaccination can effectively suppress tumor angiogenesis and lymphangiogenesis by reducing stroma-associated expression of pro-angiogenesis growth factors and cytokines.

## Discussion

**Figure 6:**
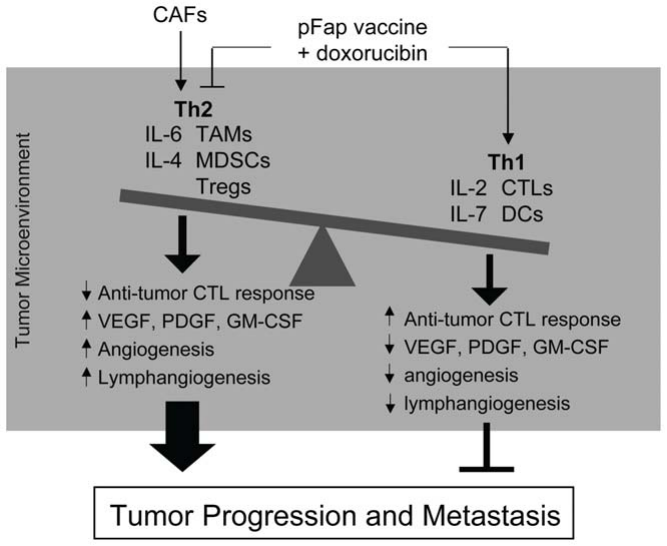
Our combination therapy exerts its anti-metastatic effects through modulation of the immune tumor microenvironment. Cancer associated fibroblasts (CAFs) promote tumor growth and metastasis by recruiting tumor associated macrophages (TAMS), myeloid derived suppressor cells (MDSCs) and T regulatory cells (Tregs) and promoting Th2 polarization of the tumor microenvironment. This modulation of the tumor microenvironment results in production of growth factors and cytokines that support tumor growth and metastasis by promoting angiogenesis, lymphangiogenesis, and suppression of anti-tumor immune responses. Elimination of CAFs by treatment with our pFap vaccine, in combination with doxorubicin chemotherapy, modulates the immune tumor microenvironment and shifts polarization from Th2 to Th1, characterized by an increase in dendritic cells (DCs) and cytotoxic T lymphocytes (CTLs) and Th1 cytokine expression. As a result, tumor angiogenesis and lymphangiogenesis are reduced and anti-tumor immune responses are enhanced leading to suppression of spontaneous metastasis of 4T1 breast cancer cells.

Our findings reported here demonstrate that CAFs promote tumor growth and metastasis through their role as key modulators of immune polarization in the TME (Figure 6). We demonstrated that elimination of CAFs *in vivo* markedly suppressed the recruitment of pro-tumor immune cells and correlated with an increase in Th1 cytokine expression in the TME. Additionally, pFap vaccination reduced stroma-associated expression of pro-angiogenesis factors and effectively suppressed tumor angiogenesis and lymphangiogenesis. Finally, the shift in Th2 to Th1 cytokine polarization induced by pFap vaccination significantly enhanced the anti-metastatic effects of doxorubicin chemotherapy. Collectively, this report adds new information to the growing evidence supporting an integral role for the TME in promoting cancer progression and re-enforces the validity and importance of CAFs as a therapeutic target for breast cancer.

These conclusions are further supported by reports of others indicating that cells in the TME influence tumor progression and metastasis through production of growth factors and cytokines that modulate these processes [6], [17]. Importantly in this regard, treatment with our combination therapy mediated a transition in the TME from a Th2 to Th1 polarization through reductions in IL-6 and IL-4 and increases in IL-2 and IL-7 cytokine expression. This finding is particularly significant since increased levels of IL-6 in tissues and serum has been shown to correlate with poor prognosis in breast cancer patients [18]. However, what effects our combination therapy has on expression levels of such cytokines as IL-6 in the serum of tumor bearing mice in our model remains to be determined. Regardless, in the context of the TME, IL-6 mediated cross-talk between stromal fibroblasts and monocytes was shown to promote the differentiation of monocytes into macrophages at the expense of DCs [19]. Additionally, receptors for IL-4 are expressed by many human tumor types and have been shown to promote the proliferation of tumor cells [20]. In contrast, Th1 cytokines IL-2 and IL-7 are potent stimulators of T-cell cytotoxic activity and of memory CD8^+^ T cell survival, respectively, both of which are critical for effective tumor immunotherapy [21], [22]. Intriguingly, splenocytes isolated from tumor bearing mice treated with our combination therapy showed an enhanced anti-tumor CTL response when exposed to irradiated 4T1 tumor cells *ex vivo*, thus demonstrating improved T-cell memory.

Importantly, Th2 cytokine polarization is also commonly associated with an angiogenesis phenotype [23], which is critical for sustained tumor growth and promotes dissemination of tumor cells [24]. Significantly, we found that expression of Vegf, Pdgfc, and GM-CSF in primary tumors was markedly reduced at both the mRNA and protein levels by treatment with our combination therapy. In this setting, paracrine VEGF signaling between tumor cells and the tumor stroma was reported to contribute to the recruitment of endothelial and lymphatic progenitors required for tumor angiogenesis and lymphangiogenesis [7]. Intriguingly, increased Pdgfc expression by CAFs caused resistance to anti-VEGF therapy in EL4 lymphoma [25], a relevant finding since our combination therapy also significantly decreased mRNA and protein expression of both Vegf and Pdgfc by the tumor stroma. Likewise, Pdgfc participated in a paracrine signaling network that accelerated tumor growth through recruitment of reactive stromal cells in malignant melanoma [26]. Additionally, GM-CSF also promotes migration and proliferation of endothelial cells [27].

We could further demonstrate that in primary tumors, pFap vaccination markedly suppressed recruitment of TAMs, MDSCs, and Tregs to the TME. These three cell types have all been identified in human malignancies and their pro-tumor and immune suppressive effects are well documented [28], [29], [30], [31], [32]. Additionally, both TAMS and MDSCs can promote the survival and invasiveness of cancer cells through secretion of growth factors and proteases [32], [33]. In contrast, Tregs directly inhibit the activation of CD8^+^ cytotoxic T cells thus reducing effective killing of tumor cells [34], [35].

Conversely, we found that pFap vaccination markedly enhanced doxorubicin-induced recruitment of DCs and CD8^+^ T cells to the TME. In this regard, DCs are of particular importance since they have been shown to directly stimulate CTLs by cross presentation of apoptotic tumor cell antigens [36], [37]. Intriguingly, doxorubicin itself can also induce immunogenic cell death by a process that depends on the phagocytic uptake of apoptotic tumor cells by local DCs and subsequent priming of CD8^+^ T cells [38], [39], [40]. Importantly, our combination therapy markedly increased the presence of DCs and activated CD8^+^ T cells in primary tumors.

Based on our findings, the anti-metastatic effects of our combination therapy are attributable to remodeling of the TME as a result of CAF clearance in addition to killing of tumor cells. As illustrated in Figure 6, the resulting modulation of the immune cell and cytokine milieu in the TME ultimately tips the balance in favor of an anti-tumor response. This reasoning could explain why maximum anti-metastatic effects are achieved when both CAFs and tumor cells are targeted for elimination.

This conclusion holds important implications for future applications of immunotherapy to treat human malignancies. In this regard, tumor cells are inherently immunogenic due to expression of tumor-associated antigens (TAAs) that can be targeted by the host immune system through vaccination with TAAs [35], [41]. Although many studies have successfully proven this concept with *in vitro* cell-based assays, the same efficacy for TAA vaccination in the clinical setting has unfortunately not yet materialized [42]. Based on our findings here, this dichotomy could be due, in part, to CAF-mediated modulation of the immune TME *in vivo*, which is excluded in *ex vivo* manipulations. Therefore, it would be intriguing to assess in the future whether our combination therapy could improve tumor immunosurveillance due to TAA vaccination *in vivo*. Regardless, our findings raise the potential that the TME could be optimized for anti-tumor effects of TAA-based immunotherapy in a clinical setting by prior treatment with our combination therapy.

## Materials and Methods

### Animals, Bacterial Strains, and Cell Lines

Animals were housed in a facility approved by the Association for Assessment and Accreditation of Laboratory Animal Care International. All animal experiments and protocols were performed according to the NIH Guide for the Care and Use of Laboratory Animals and approved by The Scripps Research Institute Animal Care Committee. Balb/c mice were purchased from The Scripps Research Institute Rodent Breeding Facility. The double attenuated S. t*yphimurium* strain RE88 (adroA-dam-) is maintained in our laboratory. Cloning and expression of cDNAs encoding murine FAP (provided by J.D. Cheng, Department of Medical Oncology, Fox Chase Cancer Center, Philadelphia, Pennsylvania, USA) in S. *typhimurium* was described previously [9]. The 4T1 murine breast carcinoma cell line was kindly provided by Suzanne Ostrand-Rosenberg (University of Maryland, College Park, Maryland, USA) and cultured in RPMI-1640 medium (ATCC) supplemented with 10% fetal bovine serum and 1% sodium bicarbonate.

### Tumor Cell Challenge, Immunization, and Doxorubicin Treatment

For orthotopic tumor cell challenge, the inguinal mammary fat pads of 3 week old female Balb/c mice were first cleared of host epithelium as previously described [43]. Mice were then challenged with 5×10̂3 4T1 tumor cells by orthotopic injection. Immunization with S. *typhimurium* in a prophylactic setting has been previously described [9]. For immunization in a therapeutic setting, mice were first challenged orthotopically and primary tumors were removed 22 d later. Animals were vaccinated twice at 2 d intervals 4 d prior to tumor resection, and then three times at 5 d intervals beginning 2 d after tumor resection. Doxorubicin (Sigma-Aldrich) was administered by i.v. injection (10 mg/kg), 3 times at 5 d intervals. For experiments performed in either prophylactic or therapeutic setting, doxorubicin treatment began 5 d after orthotopic challenge and 2 d after tumor resection, respectively. We used 8 mice per treatment group. Tumor dimensions were measured twice-weekly using calipers. Tumor volume was calculated as previously described [9].

### LCDM and QRT-PCR

Primary tumors were immediately frozen into O.C.T. Compound (Tissue-Tek) and stored at −80°C. Tumor sections, 8 µm thick, were stained with a HistoGene LCM Frozen Section Staining Kit (Arcturus) and tissues captured with an Arcturus PixCell II Laser Capture Microdissection microscope (Arcturus), according to the manufacturer's protocol. Total RNA was isolated with the PicoPure RNA Isolation Kit (Arcturus) and cDNA generated using the QuantiTect Whole Transcriptome Kit (Invitrogen). QRT-PCR was performed with primer sets from RealTimePrimers.com according to the manufacturer's protocol. Gene expression was normalized to β-actin. Results represent data combined from three different experiments.

### Immunohistochemistry

Frozen tumor sections were fixed in cold acetone and blocked in 10% normal goat serum prior to incubation with antibody: rat-αmouse CD8 (AbD Serotec), rat-αmouse DCs (BD Pharmingen), rat-αmouse F4/80 (AbD Serotec), and rat-αmouse CD4 (BD Pharmingen), α-mouse FOXP3 (Santa Cruz Biotechnology), rabbit-αmouse LYVE1 (Reliatech), rabbit-αmouse CD31 (BD Pharmingen), and rabbit-αmouse Caspase 3 (Santa Cruz Biotechnology). Apoptotic tumor cells were detected using the DeadEnd Colorimetric TUNEL System (Promega). For determination of percent TUNEL positive cells, a minimum of 9 different fields per group were counted at 20× magnification for TUNEL positive cells and total cell number.

### Histology of Pulmonary Metastasis

For visualization of pulmonary metastasis, lung tissue was fixed in 10% phosphate buffered. Lungs were then cut into 20 µm thick serial sections and stained with hematoxylin and eosin. The surface area of metastatic foci in lungs (n = 6 per group) was measured using ImageJ software.

### Flow Cytometry

Live primary tumor cell suspensions were isolated from tumor bearing mice and analyzed immediately for activated CD8^+^ T cells with 2-color flow cytometry using α-CD8 antibody (BD Pharmingen) and α-CD25 antibody (BD Pharmingen). Results are combined from 2 different experiments. To determine anti-tumor CTL response, splenocytes were cultured for 5 days and stimulated twice with recombinant IL-2 (50 u/ml) (PeproTech) and then incubated with 10̂6 irradiated 4T1 tumor cells for 24 hours prior to staining with α-CD8 and α-Granzyme B antibodies, according to the manufacturer's protocol (BD Pharmingen), and analysis using flow cytometry. Results are representative of 2 separate experiments.

### Western Blots

Tumor whole cell extracts were generated as previously described [44] and α-VEGF, α-GM-CSF, α-Pdgfc, αIL-2, αIL-7, αIL-4, and α-actin (all from Santa Cruz Biotechnology), and αIL-6 (R&D Systems) antibodies were used.

### Statistical Analysis

Statistical analysis was done with Prism 5.0 (GraphPad Software) and Excell (Microsoft) software. Statistical significance was determined by student's *t* test (significance when p<0.05). Survival curves were analyzed by the Kaplan-Meier survival method, and statistical significance was determined using the log-rank test (significance when p<0.05).
